# Primary malignant melanoma of the esophagogastric junction: A case report

**DOI:** 10.1097/MD.0000000000026467

**Published:** 2021-06-25

**Authors:** Yu-Ming Chu, Chih-Sheng Hung, Ching-Shui Huang

**Affiliations:** a Division of Digestive Medicine, Department of Internal Medicine, Cathay General Hospital; b School of Medicine, Fu-Jen Catholic University, New Taipei City; c Division of General Surgery, Department of Surgery, Cathay General Hospital, Taipei City, Taiwan.

**Keywords:** esophageal tumor, esophagogastric junction, primary malignant melanoma

## Abstract

**Rationale:**

Most gastrointestinal melanomas are metastatic from an oculocutaneous primary lesion; however, primary gastrointestinal melanomas have been found in all levels of the gastrointestinal tract. We present the case of Primary malignant melanoma of the esophagus and discuss the diagnostic methods, differentiation from metastatic lesions and treatment options.

**Patient concerns:**

A 78-year-old male patient presented with fresh blood vomiting and tarry stools for 1 day.

**Diagnoses:**

Esophagogastroduodenoscopy of this patient revealed a tumor ∼4 cm in size at the cardia side of the esophagogastric junction with dark-red and gray pigmentation. Immunohistochemical stains of the biopsy specimens were positive for S-100 and HMB-45, which are specific markers of melanoma.

**Interventions:**

Laparotomy with proximal gastrectomy was performed by the surgeon. Histological examination of the surgical specimen revealed the tumor arose from the distal esophagus with invasion of the proximal stomach. Primary malignant melanoma of the esophagus was diagnosed after a full skin and ophthalmic examination and positron emission tomography, which revealed no lesions elsewhere in the body.

**Outcomes:**

No tumor recurrence was noted at the 1-year follow-up.

**Lessons:**

Primary malignant melanoma of the esophagus is an extremely rare but highly aggressive tumor. The special pattern of pigmentation should be recognized while performing endoscopy. Early detection and radical resection of the tumor are critical to ensure favorable outcomes.

## 1 Introduction

Primary malignant melanoma of the esophagus (PMME) is an extremely rare and aggressive disease, accounting for only 0.1% to 0.2% of all tumors of the esophagus.^[1,2]^ The diagnosis of PMME should be based on the combination of morphological examination, pathological examination, and immunohistochemistry.^[3]^ The main treatment of PMME remains radical resection of the tumor. However, the optimal adjuvant therapies for PMME have not yet been established.^[3]^

We report a case of malignant melanoma of the esophagogastric junction, originating from the distal esophagus but situated in the gastric cardia. The patient has remained disease free for 1 year since the surgery.

## 2 Case report

**Figure 1:**
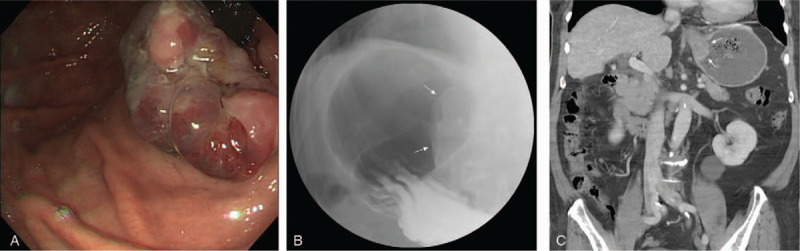
(A) EGD revealed a tumor at the cardia side of the esophagogastric junction with dark-red and gray pigmentation. (B) Upper gastrointestinal series indicated a nearly 4-cm submucosal mass at the gastric cardia. (C) CT indicated a mass with rim enhancement. CT = computed tomography, EGD = Esophagogastroduodenoscopy.

A 78-year-old man was admitted for fresh blood vomiting and tarry stools lasting 1 day. The patient had hypertension and had received cholecystectomy many years previously. He denied abdominal pain but mentioned epigastric fullness and decreased appetite in the 1 month prior. His weight had also decreased by ∼5 kg in the preceding month. Physical examination showed only pale conjunctiva. Laboratory data revealed normocytic anemia with a hemoglobin level of 9.6 g/dL and mean corpuscular volume of 85.3 fL. The platelet count and coagulation test result were all within normal range. Esophagogastroduodenoscopy (EGD) revealed a tumor ∼4 cm in size at the cardia side of the Esophagogastric junction and that had dark-red and gray pigmentation (Fig. 1A). No specific mucosal pattern was detected under white light and narrow band imaging; subepithelial tumor or undifferentiated cancer was suspected, and a biopsy was performed. The upper gastrointestinal series indicated a nearly 4-cm submucosal mass at the gastric cardia (Fig. 1B). Computed tomography (CT) indicated a nearly 4-cm mass at the gastric cardia with rim enhancement (Fig. 1C). The results for tumor markers, including carcinoembryonic antigen and carbohydrate antigen 19–9, were all negative.

**Figure 2:**
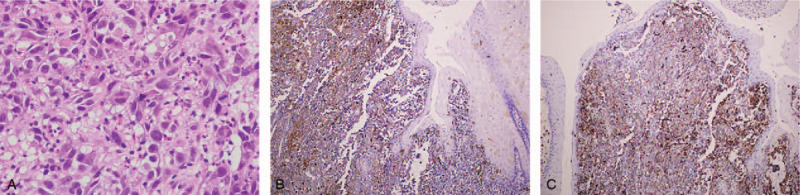
(A) Biopsy specimens showed pleomorphic polyhedral or fusiform cells with a high nucleus-to-cytoplasm ratio. In immunohistochemical stains, tumor cells were positive for S-100 (B) and HMB-45 (C).

**Figure 3:**
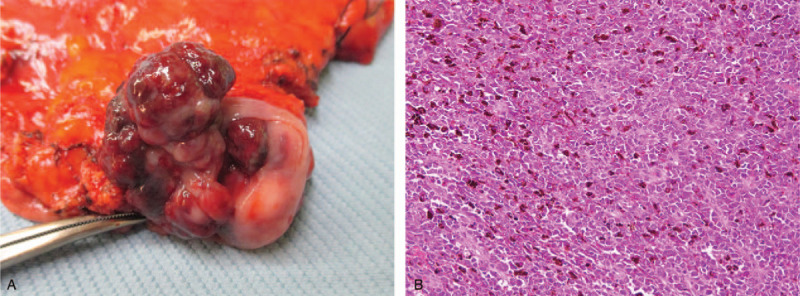
(A) Surgical specimen was a polypoid tumor with black and gray pigmentation and surface ulceration. (B) Histological exam revealed large pleomorphic neoplastic cells with melanin pigments.

Biopsy specimens obtained from the Esophagogastric junction comprised pleomorphic polyhedral or fusiform cells with a high nucleus-to-cytoplasm ratio (Fig. 2A). In immunohistochemical stains, tumor cells were shown to be positive for S-100 (Fig. 2B), and HMB-45 (Fig. 2C), which are specific markers of melanoma, and to be negative for cytokeratin, p40, and leukocyte common antigen. These results indicated malignant melanoma. In a full skin and ophthalmic examination, no primary lesion was found. Positron emission tomography was performed and revealed an area of increased radioactivity in the stomach but not elsewhere in the body. These results indicated primary malignant melanoma of the esophagogastric junction. Laparotomy with proximal gastrectomy was performed by the surgeon. The specimen was a polypoid tumor, 5.5 cm × 3.7 cm in size, with black and gray pigmentation and surface ulceration, and that was situated in the upper stomach (Fig. 3A). Histological examination results were compatible with malignant melanoma with melanin pigment present (Fig. 3B), arising from the distal esophagus with invasion of the proximal stomach. The tumor was mainly located in the submucosa and focally to the superficial muscularis propria. Detailed pathological examination revealed focal junctional activities in the esophageal squamous epithelium. No lymph node metastasis was observed. Genetic study revealed absence of the BRAF mutation. Because the surgical margin was free and no lymph node or other organ metastasis was found, we closely followed up the patient without performing chemotherapy or radiotherapy. Contrast-enhanced CT examinations were performed at 3 and 9 months, and EGD was performed 1 year after the operation. No tumor recurrence was noted at the 1-year follow-up.

## 3 Discussion

Malignant melanoma of the gastrointestinal tract is usually a metastasis from a primary cutaneous source. PMME is extremely rare, accounting for only 0.1% to 0.2% of all tumors of the esophagus.^[1,2]^ PMME most commonly occurs in men, with a male-to-female ratio of ∼2:1, and the average age of onset is 60.5 years. The tumor is usually located in the middle and lower third of the esophagus (76.2%).^[4,5]^ In our case, we observed no tumors in the esophagus lumen; the main tumor was in the gastric cardia, but the final pathological report indicated the tumor arose from the distal esophagus with invasion of the proximal stomach.

The endoscopic finding of PMME is usually a well-circumscribed, solid, polypoid tumor with black or purple pigmentation on the surface; sometimes accompanied by ulcers and bleeding.^[3,5]^ In contrast, metastatic melanoma usually has multiple nodular lesions and may be distributed in various parts of the gastrointestinal tract.^[6]^ However, some PMMEs present as a flat lesion^[7]^ or as multinodular lesions that are difficult to distinguish from metastatic lesions.^[8,9]^ Surface pigmentation is characteristic of gastrointestinal melanoma, but some melanomas lack melanin—the so-called amelanotic melanomas; these account for 10% to 25% of all PMMEs and are extremely difficult to distinguish from other tumor types.^[10]^ An accurate preoperative diagnosis of primary malignant melanoma is difficult to make from a biopsy specimen because the biopsy results are easily misinterpreted as indicating undifferentiated carcinoma. Repeated endoscopic biopsy may be required.^[11]^ A definite diagnosis of melanoma depends on an immunohistochemical examination showing positive results for S-100 protein, HMB-45, and neuron-specific enolase.^[3]^

Histologic criteria for primary malignant melanoma of the esophagus have been developed by Allen and Spitz^[12]^; they emphasize the presence of junctional changes, which are defined as some nests of melanocytes with varying degrees of atypia at the mucosal–submucosal junction adjacent to the tumor mass. Unfortunately, this major criterion is met in only ∼40% of cases because if the tumor grows rapidly, the adjacent junctional changes may not be observed.^[3,13]^ Suggestive diagnostic criteria of primary gastrointestinal melanoma include lack of a concurrent lesion in the skin or other organ and absence of a history of removal of melanoma or atypical melanocytic lesion.^[14]^

No standard tumor, node, metastasis (TNM) staging system currently exists for PMME. According to the eighth edition of the American Joint Committee on Cancer (AJCC) staging criteria, patients presenting with distant visceral metastases, without a detectable primary tumor, and receiving a diagnosis of stage IV disease have similar or slightly better outcomes than those with metastatic disease and a known primary site.^[15]^ In some studies, initial TNM stage of PMME according to the AJCC classification for esophageal cancer was significantly related to overall survival.^[2,16]^ One study conducted in China including 20 cases reported that the 5-year survival rate of PMME was 16.9%, which is consistent with previous reports of 4% to 37%.^[4]^ The incidence of metastasis to the regional lymph nodes is high at 40% to 80% at the initial diagnosis.^[3,17]^ Tumor invasion of the submucosal layer is associated with higher incidence of lymph node metastasis, which has a poor prognosis.^[4,18]^

No standard guideline exists for the treatment of primary gastrointestinal melanoma. Early diagnosis, radical surgical excision, and aggressive lymph node dissection have been beneficial for accurate staging and better clinical outcomes.^[18,19]^ The traditional adjuvant therapies after surgery for patients with PMME are chemotherapy (e.g., dacarbazine) and radiotherapy. These therapies do not exhibit real effectiveness, but a few authors have reported sporadic cases of favorable responses.^[3]^ Over the past decade, the introduction of novel therapies has drastically improved the survival of patients with advanced melanoma, and these therapies are broadly grouped into immune checkpoint inhibitors (immunotherapy) and BRAF or MEK inhibitors (targeted therapy).^[20]^ A nationwide study revealed that marked improvements in overall survival were associated with the use of targeted therapy and immunotherapy in patients with stage IV melanoma with unknown primary site.^[20]^ These findings could be used in clinical practice and the treatment of PMME, but more studies are required to prove the benefit.

In conclusion, PMME is an extremely rare but highly aggressive tumor. The special pattern of pigmentation should be recognized while performing endoscopy. Diagnosis of PMME requires careful pathological examination and exclusion of other possible origins in the whole body. Early detection and radical resection of the tumor are critical to ensure favorable outcomes. The effect of adjuvant chemotherapy and radiotherapy is uncertain. Novel therapies—immunotherapy and targeted therapy—may improve the overall survival in this disease.

## Author contributions

**Conceptualization:** Chih-Sheng Hung, Ching-Shui Huang.

**Data curation:** Chih-Sheng Hung, Ching-Shui Huang.

**Investigation:** Yu-Ming Chu.

**Supervision:** Chih-Sheng Hung, Ching-Shui Huang.

**Writing – original draft:** Yu-Ming Chu.

**Writing – review & editing:** Yu-Ming Chu.
